# Association of Dipstick Proteinuria with Long-Term Mortality among Patients with Hypertensive Crisis in the Emergency Department

**DOI:** 10.3390/jpm12060971

**Published:** 2022-06-14

**Authors:** Byung Sik Kim, Mi-Yeon Yu, Jin-Kyu Park, Jinho Shin, Jeong-Hun Shin

**Affiliations:** 1 Division of Cardiology, Department of Internal Medicine, Hanyang University Guri Hospital, Hanyang University College of Medicine, Guri-si 11923, Korea; fish3777@hanmail.net; 2 Division of Nephrology, Department of Internal Medicine, Hanyang University Guri Hospital, Hanyang University College of Medicine, Guri-si 11923, Korea; pure8203@gmail.com; 3 Division of Cardiology, Department of Internal Medicine, Hanyang University Seoul Hospital, Hanyang University College of Medicine, Seoul 04763, Korea; cardiohy@hanyang.ac.kr (J.-K.P.); jhs2003@hanyang.ac.kr (J.S.)

**Keywords:** proteinuria, hypertensive crisis, emergency department, mortality

## Abstract

Proteinuria, frequently observed in hypertensive crisis, is a risk factor for cardiovascular and all-cause mortality in patients with hypertension. Here we investigated the association between proteinuria and all-cause mortality in patients with a hypertensive crisis in the emergency department (ED). This retrospective study included patients admitted to the ED of a tertiary referral center between 2016 and 2019 with hypertensive crisis (systolic blood pressure ≥ 180 mmHg or diastolic blood pressure ≥ 110 mmHg); 3599 patients with an assay for proteinuria were included in this study. Proteinuria was defined as a trace or more protein on a urine dipstick test. Proteinuria was present in 1964 (54.6%) of 3599 patients. At 3 years, crude all-cause mortality rates were 10.8% for patients with negative proteinuria, 21.7% for those with trace proteinuria, 29.0% for those with proteinuria (1+), 32.0% for those with proteinuria (2+), and 35.4% for those with proteinuria (≥3+). After adjusting for age, sex, blood pressure, and comorbid conditions, the hazard ratio (95% confidence interval) for dipstick proteinuria was 1.91 (1.53–2.37) for those with trace proteinuria, 2.32 (1.85–2.91) for those with proteinuria (1+), 2.40 (1.86–3.10) for those with proteinuria (2+), and 2.40 (1.78–3.24) for those with proteinuria (≥3+) compared to the reference of negative proteinuria. In patients with hypertensive crisis, dipstick proteinuria was a significant predictor of all-cause mortality, and the risk of all-cause mortality increased in a dose-dependent manner according to its degree. Moreover, even trace proteinuria was associated with an increased risk of mortality. The dipstick urine test could be used as a simple and useful method for risk assessment of all-cause mortality in patients with hypertensive crisis.

## 1. Introduction

Hypertensive crisis presents as an abrupt elevation of blood pressure (BP) associated with or without hypertension-mediated organ damage (HMOD) [1]. Approximately 1–2% of patients with hypertension experience acute and severe elevation in blood pressure (BP), termed a “hypertensive crisis” [2]. Treatment for hypertension and survival has improved dramatically over the past decades, but patients with hypertensive crisis remain at increased risk of cardiovascular events and mortality compared with hypertensive patients who did not experience an emergency [3,4,5,6].

Proteinuria, which is a marker of renal damage and a marker of HMOD, is associated with an increased risk of cardiovascular disease and all-cause and cardiovascular mortality [7]. The diagnosis of hypertension-induced renal damage is based on the finding of reduced kidney function or the detection of proteinuria [8,9]. Therefore, the assessment of proteinuria is important in patients with hypertensive crisis and helps identify patients at high risk of death and consequently improve clinical outcomes. Although quantitative testing of albumin using the albumin-to-creatinine ratio in a spot urine sample is recommended to assess renal impairment in all patients with hypertension, the urine dipstick test is widely used in clinical practice and screening for the evaluation of proteinuria owing to its low cost, simplicity, rapid results, and acceptable accuracy [10,11].

Several studies have demonstrated the association between proteinuria and mortality outcomes in patients with hypertension and the general population [7,8,9,12]. However, data concerning the association between dipstick proteinuria and mortality and their prognostic significance are scarce in patients with hypertensive crisis. Therefore, we aimed to assess the association between dipstick proteinuria and mortality in patients with hypertensive crisis visiting the emergency department (ED).

## 2. Materials and Methods

### 2.1. Study Participants

**Figure 1:**
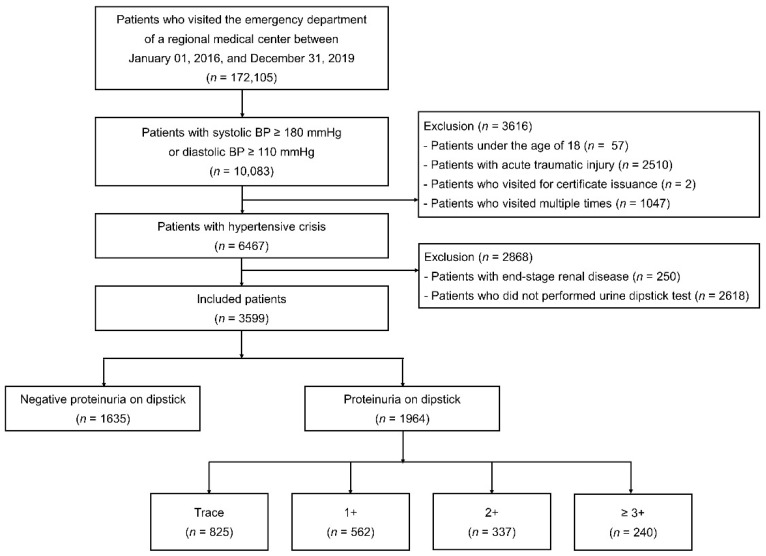
Flow diagram illustrating patients with a hypertensive crisis who were included in this study. BP, blood pressure.

This observational study was conducted at a single regional emergency medical center affiliated with the Academic University Hospital in Guri-si, Gyeonggi-do, Korea. Detailed information about the study design and definitions used in the study have been previously published [6,13]. In brief, we reviewed the medical records of 6467 patients with a hypertensive crisis who visited the ED between 2016 and 2019. Hypertensive crisis was defined as systolic blood pressure (SBP) ≥ 180 mmHg or diastolic blood pressure (DBP) ≥ 110 mmHg. Patients with acute trauma or those who had visited for obtaining a medical certificate were excluded. Only data from the first visit were included for patients who visited the ED multiple times. Among the 6467 patients with hypertensive crisis, patients with end-stage renal disease who were on dialysis and those who had no dipstick urine analysis data during the ED visit were excluded. Finally, 3599 patients were included in this study (Figure 1).

### 2.2. Proteinuria Measurement

Urine protein levels were measured using AUTION dipsticks (ARKRAY. Inc., Kyoto, Japan) using a fully automated urine chemistry analyzer (AX-4030, ARKRAY. Inc., Kyoto, Japan) during the index visit, which was part of the routine care for admitted patients in our hospital throughout the study period. The dipstick reader and strips are known to be reliable based on the database of the Korean Association of External Quality Assessment Service [14]. Using an automated urine dipstick analyzer, the degree of proteinuria was interpreted as negative, trace (approximately 15 mg/dL), 1+ (30 mg/dL), 2+ (100 mg/dL), 3+ (300 mg/dL), and 4+ (1000 mg/dL), according to the manufacturer’s instructions. For the analyses, results were categorized as negative, trace, 1+, 2+, and ≥3+.

### 2.3. Data Collection and Outcomes

Data were collected using electronic medical records by trained data collectors under the supervision of the principal investigator. The collected data included demographic and clinical characteristics, cardiovascular risk factors, BP, previous medical history, presence and types of acute HMOD, and diagnostic test findings at the index visit to the ED. In addition, events during the hospitalization and follow-up periods (e.g., admission, discharge, ED revisits, readmission, and death) were collected. In the ED, BP was measured on the brachial artery using an automated BP machine, Spot Vital Signs LXi (Welch Allyn, Skaneateles Falls, New York, NY, USA). Cardiomegaly on chest radiography was diagnosed when the ratio between the maximal horizontal cardiac diameter and the maximal horizontal inner thoracic cage diameter was >0.5 [15]. Left ventricular hypertrophy (LVH) on electrocardiography (ECG) was diagnosed when it satisfied either the Cornell voltage criterion (the amplitude of R in aVL plus the amplitude of S or QS complex in V3 with a cutoff of >2.8 mV in men and >2.0 mV in women) or the Sokolow–Lyon criterion (the amplitude of S in V1 plus the amplitude of R in V5 or V6 ≥ 3.5 mV) [16].

The included patients were followed up until death from any cause or on March 2021 (study endpoint), whichever came first. The incidence and timing of mortality were obtained from the National Health Insurance Service in South Korea.

### 2.4. Statistical Analysis

Continuous variables were presented as means (standard deviation, SD), whereas categorical data were presented as numbers (percentages). One-way analysis of variance was performed to compare baseline characteristics according to the degree of proteinuria for continuous variables. The Cochran-Mantel-Haenszel test was performed to show the trend of the categorical data according to the degree of proteinuria. The Kaplan–Meier method and log-rank test were used to compare cumulative survival probability. 

Using a Cox proportional hazard model and negative proteinuria as a reference, hazard ratios (HRs) and 95% confidence intervals (CIs) for 3-year all-cause mortality according to the degree of proteinuria were analyzed. A multivariable Cox proportional hazard model was performed by adjusting for other clinically relevant variables, including baseline characteristics (age, sex, BP), comorbidities (hypertension, diabetes mellitus, ischemic stroke, hemorrhagic stroke, and coronary artery disease), and components of subclinical HMOD (serum creatinine, cardiomegaly on chest radiography, and LVH on ECG). Furthermore, subgroup analyses were performed on the basis of age (<65 or ≥65 years), sex, presence of acute HMOD, estimated glomerular filtration rate (eGFR) (<60 or ≥60 mL/min/1.73 m^2^), and presence of diabetes mellitus by constructing a multivariable Cox model with adjustment for the same variables. All tests were two-tailed, and the null hypothesis was rejected for a value of *p* < 0.05. All analyses were performed using the Statistical Analysis Software package (SAS version 9.4; SAS Institute, Cary, NC, USA).

## 3. Results

### 3.1. Baseline Characteristics

A total of 3599 patients were included in this study. The most common principal diagnosis at discharge was cardiovascular disorders, such as acute heart failure, angina pectoris, and acute myocardial infarction (28.0%). Other principal diagnoses included neurologic disorders (27.5%; ischemic stroke, hemorrhagic stroke, and seizures), and infectious diseases (12.9%) (Supplementary Table S1). Follow-up data for up to 5.2 years were analyzed and the median follow-up period was 2.9 years (interquartile range, 1.9–4.0 years). The baseline characteristics of the patients according to the degree of proteinuria are shown in Table 1. Among these patients, 1964 (54.6%) had proteinuria, 825 had trace proteinuria, 562 had proteinuria (1+), 337 had proteinuria (2+), and 240 had proteinuria (≥3+). Patients with higher proteinuria were older, had higher SBP, and had more comorbidities, such as hypertension, diabetes mellitus, ischemic or hemorrhagic stroke, coronary artery disease, heart failure, and chronic kidney disease. A significantly increasing trend according to the degree of proteinuria was observed for serum creatinine, eGFR, troponin-I, and B-type natriuretic peptide (BNP) levels. The frequency of cardiomegaly on chest radiography and LVH on ECG were more frequently observed along with the severity of dipstick proteinuria. The presence of acute HMOD also significantly increased along with a higher degree of dipstick proteinuria.

### 3.2. Outcomes of the Index Visit and During the Follow-Up Period

Overall, 2199 (61.1%) patients were admitted and 1108 (30.8%) patients were discharged: 288 (8.0%) patients were discharged against medical advice, and 4 (0.1%) patients died in the ED. The admission rate increased with the degree of proteinuria, while the discharge rate decreased with the degree of proteinuria (both *p* <0.001). There were no differences between groups in the rate of ED revisits and rate of readmission within 1 month; however, rates of ED revisit and rates of readmission within 3 months and 1 year increased along with the degree of proteinuria (Table 2).

**Figure 2:**
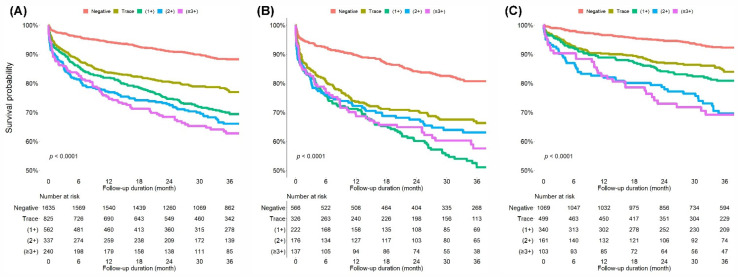
Kaplan–Meier curves display the survival probability of all-cause mortality according to the degree of dipstick proteinuria. (**A**) All patients. (**B**) Patients with acute HMOD. (**C**) Patients without acute HMOD. HMOD, hypertension-mediated organ damage.

During the follow-up period, 3-year all-cause mortality increased along with an increase in proteinuria in all patients (Figure 2A). At 3 years, crude all-cause mortality rates were 10.8% for patients with negative proteinuria, 21.7% for those with trace proteinuria, 29.0% for those with proteinuria (1+), 32.0% for those with proteinuria (2+), and 35.4% for those with proteinuria (≥3+). Kaplan–Meier curves for the cumulative death-free survival probability are presented in Figure 2B for patients with acute HMOD and Figure 2C for patients without acute HMOD. The higher the level of proteinuria, the lower the cumulative survival probability in patients without acute HMOD (log-rank *p* < 0.001). In contrast, this trend was not prominent in patients with acute HMOD. However, the cumulative survival probability was significantly lower in patients with proteinuria than in those without proteinuria. 

To examine the independent association between proteinuria and all-cause mortality, we performed a Cox regression analysis (Table 3). In the unadjusted model, trace proteinuria, proteinuria (1+), proteinuria (2+), and proteinuria (≥3+) were significantly associated with 3-year all-cause mortality, and these associations were pronounced as proteinuria degree increased. After adjusting for sex, age, BP, and comorbidities and components of subclinical HMOD, the HR (95% CI) for dipstick proteinuria was 1.91 (1.53–2.37) for those with trace proteinuria, 2.32 (1.85–2.91) for those with proteinuria (1+), 2.40 (1.86–3.10) for those with proteinuria (2+), 2.40 (1.78–3.24) for those with proteinuria (≥3+) compared with the reference of negative proteinuria (−). Adjustment for covariates weakened the association between proteinuria levels and mortality risk but maintained the statistical significance of mortality risk after the adjustments. 

**Figure 3:**
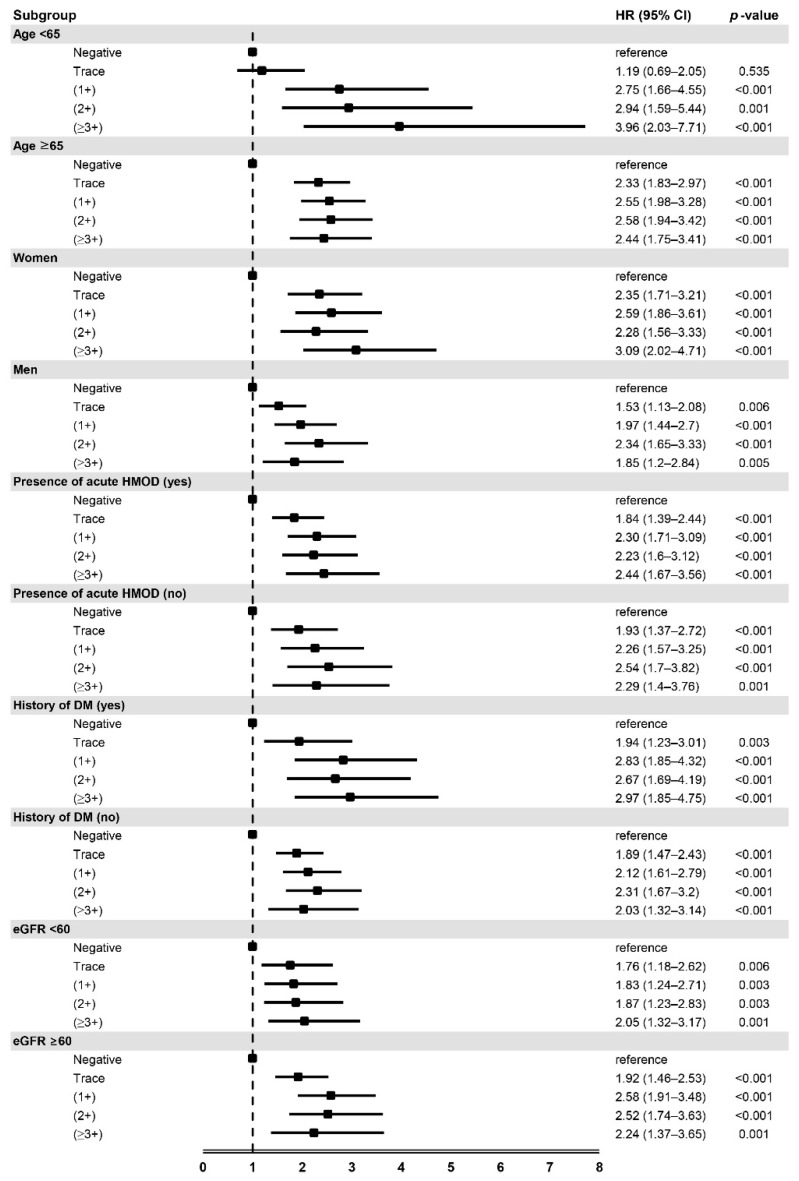
Adjusted hazard ratios of 3-year all-cause mortality according to the degree of dipstick proteinuria in subgroups. HR, hazard ratio; CI, confidence interval; HMOD, hypertension-mediated organ damage; DM, diabetes mellitus; eGFR, estimated glomerular filtration rate. Hazard ratios were adjusted by age, sex, systolic blood pressure, diastolic blood pressure, comorbidities (hypertension, diabetes mellitus, ischemic stroke, hemorrhagic stroke, and coronary artery disease), and components of HMOD (serum creatinine, cardiomegaly in chest radiography, and left ventricular hypertrophy in electrocardiography).

We performed a subgroup analysis stratified by the covariates, including age (<65 or ≥65 years), sex, presence of acute HMOD, eGFR (<60 or ≥60 mL/min/1.73 m^2^), and presence of diabetes mellitus. The adjusted HRs and 95% CIs for 3-year all-cause mortality of the four categories (trace proteinuria, proteinuria (1+), proteinuria (2+), and proteinuria (≥3+)) were similarly higher than negative proteinuria in all subgroups, except that trace proteinuria in patients aged <65 years were not significantly associated with an increased risk of 3-year all-cause mortality (Figure 3).

## 4. Discussion

This study investigated the association between dipstick proteinuria and the risk of long-term mortality in patients with hypertensive crisis. Patients with proteinuria had a higher risk of mortality independent of other risk factors, including age, sex, BP, comorbidities, and components of subclinical HMOD. The risk of all-cause mortality increased in a dose-dependent manner according to the degree of dipstick proteinuria. Our study findings suggest that the dipstick proteinuria test may be valuable for identifying patients with a hypertensive crisis who are at high risk of mortality.

Hypertension can be both a cause and a complication of chronic kidney disease. Moreover, uncontrolled BP, which can lead to rapid renal impairment and even mild reductions in kidney function, is associated with a higher risk of cardiovascular events in hypertensive patients [17,18]. Therefore, diagnosis of hypertensive nephropathy or identification of patients who are at high risk of developing renal damage is vital for the effective management of patients with hypertension. In this regard, guidelines for the management of hypertension recommend routine assessment of hypertension-induced renal damage by using simple renal function parameters (serum creatinine and eGFR) together with the investigation of albuminuria (dipstick or urinary albumin creatinine ratio) [10,19,20]. 

Proteinuria is a marker of kidney disease and is associated with cardiovascular disease and mortality in the general population. In addition, studies in hypertensive populations indicate that proteinuria is an adverse prognostic indicator of clinical cardiovascular disease and mortality [21,22]. Moreover, proteinuria was associated with all-cause mortality in patients with myocardial infarction [23], heart failure [24], and diabetes or pre-diabetes [25]. In this study, we demonstrated that trace proteinuria showed an HR of 1.91 (95% CI 1.53–2.37) and proteinuria (≥3+) showed an HR of 2.40 (95% CI 1.78–3.24) for all-cause mortality using a fully adjusted model; these increased risks were consistent in most subgroups. This highlights the importance of evaluating proteinuria even in patients with hypertensive crisis.

Several potential mechanisms may help explain the relationship between proteinuria and all-cause mortality in patients with hypertensive crisis. First, patients with a higher degree of proteinuria had a higher frequency of acute HMOD. Second, patients with a higher degree of proteinuria tended to have more comorbidities and cardiovascular risk factors such as hypertension, diabetes mellitus, ischemic or hemorrhagic stroke, coronary artery disease, heart failure, and chronic kidney disease. Third, patients with a higher degree of proteinuria more frequently showed abnormal laboratory findings associated with subclinical HMOD, such as cardiomegaly on chest radiography, and LVH on ECG; in addition, these patients had higher levels of creatinine, troponin-I, and BNP associated with a risk of adverse cardiovascular events [26,27,28]. Moreover, the association between proteinuria and a higher risk of all-cause mortality persisted even after adjusting for confounding variables. In addition, proteinuria may reflect generalized vascular dysfunction and is associated with a higher susceptibility to cardiovascular events [29]. Another explanation may be that genetically elevated proteinuria, especially albuminuria, led to higher BP, and bidirectional effects between albuminuria and BP may contribute to an increased risk of cardiovascular disease [30]. Further studies are required to clarify the mechanisms linking proteinuria to the excess risk of death in patients with hypertensive crisis.

In this study, we used a urine dipstick test, which is routinely used in daily practice in the ED to assess proteinuria. There are concerns over the low sensitivity and accuracy of the urine dipstick test for the detection of proteinuria [31]. However, the urine dipstick test is widely used in clinical practice and screening for the evaluation of proteinuria owing to its low cost, ease of use, rapid results, and acceptable accuracy [32,33]. Therefore, the dipstick urine test would be helpful in clinical situations such as in EDs where routine tests require quick results or in medical environments where more accurate tests are not available. In addition, dipstick proteinuria has been reported as a predictor of mortality risk in the general population, and this prognostic significance was observed not only in higher degrees of proteinuria but also in trace proteinuria detected with the dipstick test [32,34]. Our data also showed that trace proteinuria was significantly associated with an increased risk of all-cause mortality. This result suggests that it is necessary to consider patients with hypertensive crisis who have proteinuria, even at a mild degree, as a high-risk group. These patients require appropriate treatment and close follow-up. 

Taken together, our results suggest that the identification of proteinuria by a simple dipstick urine test may be important in the management of patients with hypertensive crisis in the ED. Early detection and treatment of kidney injury can help clinical doctors determine appropriate kidney protective agents for high-risk patients. Moreover, as a result of this study, the dose-response increase in all-cause mortality risk according to the degree of proteinuria suggests that proteinuria reduction would have a beneficial effect on the prevention of mortality. Modifications in lifestyle factors and antihypertensive therapies such as angiotensin-converting enzyme inhibitors or angiotensin receptor blockers may provide an additive effect on the reduction of proteinuria [35,36,37]. Thus, further research is needed to evaluate the effect of these interventions on proteinuria reduction to aid in the prevention of cardiovascular disease and death.

Our study has a few limitations. First, it was a single-center retrospective study. Given its retrospective nature, data on the study participants’ baseline characteristics were insufficient compared with those of prospective studies. Due to the limitation of the observational design without interventions, we could not conclude whether proteinuria had a causal effect on the risk of mortality or only reflected other deteriorating conditions associated with uncontrolled hypertension. We were also unable to explain the exact mechanism of the association between proteinuria and the risk of all-cause mortality. Second, urine dipstick proteinuria was assessed using a single measurement, which may have led to a measurement error. Although this value may be inaccurate, it can better reflect the actual screening test in practice. Moreover, we collected only the diagnostic test results that were performed in the ED. Third, as we used only initial BP values and did not consider BP control in the ED or after discharge, further analysis of the outcomes according to the BP control patterns was limited. In addition, we could not obtain data on the reasons for revisits or readmissions after the index ED visits. Fourth, we could not identify the cardiovascular and renal events or cardiovascular mortality because the National Health Insurance Service data did not provide information on the causes of death. However, data regarding all-cause mortality and date of death were mathematical because they were obtained from the National Health Insurance Service, which covers the entire Korean population. Fifth, the study included data from a single center, it may not be representative of the entire population. Further research is needed to determine the optimal screenings, risk stratifications, and treatment strategies related to cardiovascular events or deaths according to dipstick proteinuria results in patients with hypertensive crisis. Finally, diagnostic tests, including the dipstick urine test, were not performed in all patients, and it is possible that more tests were performed in relatively high-risk patients than in low-risk patients. Therefore, selection bias could not be excluded from this study.

## 5. Conclusions

In conclusion, the present study demonstrated that dipstick proteinuria was an independent risk factor for long-term mortality in patients with hypertensive crisis. The risk of all-cause mortality increased proportionally with the severity of dipstick proteinuria. Moreover, even trace proteinuria was associated with an increased risk of mortality. Further, large-scale clinical studies should be conducted to investigate the exact mechanisms of proteinuria and the risk of death in patients with hypertensive crisis.

## Figures and Tables

**Table 1:** Baseline characteristics.

	All Patients (*n* = 3599)	Negative (*n* = 1635)	Dipstick Proteinuria				*p*-Value
			Trace (*n* = 825)	1+ (*n* = 562)	2+ (*n* = 337)	≥3+ (*n* = 240)	
Age, mean (SD)	63.1 (16.8)	60.9 (16.1)	63.2 (17.6)	65.0 (17.3)	67.9 (16.3)	66.7 (16.3)	<0.001
Female sex, *n* (%)	1795 (49.9)	878 (53.7)	385 (46.7)	267 (47.5)	158 (46.9)	107 (44.6)	<0.001
Medical history, *n* (%)							
Hypertension	1935 (55.0)	789 (49.2)	420 (52.2)	322 (59.0)	226 (69.3)	178 (74.8)	<0.001
Diabetes mellitus	931 (26.6)	271 (16.9)	204 (25.7)	185 (34.3)	138 (42.3)	133 (56.4)	<0.001
Dyslipidemia	357 (10.3)	168 (10.6)	81 (10.3)	47 (8.7)	31 (9.7)	30 (12.9)	0.955
Ischemic stroke	327 (9.4)	111 (7.0)	73 (9.3)	69 (12.8)	42 (13.0)	32 (13.7)	<0.001
Hemorrhagic stroke	108 (3.1)	32 (2.0)	30 (3.8)	26 (4.8)	11 (3.5)	9 (3.9)	0.008
Coronary artery disease	315 (9.1)	125 (7.9)	66 (8.4)	50 (9.3)	44 (13.7)	30 (12.9)	<0.001
Heart failure	160 (4.6)	33 (2.1)	35 (4.5)	29 (5.4)	32 (9.9)	31 (13.3)	<0.001
Chronic kidney disease	188 (5.4)	21 (1.3)	29 (3.7)	33 (6.1)	41 (12.8)	64 (27.2)	<0.001
Social history, *n* (%)							
Cigarette smoking	751 (29.1)	330 (29.0)	184 (31.4)	108 (26.2)	68 (25.9)	61 (32.3)	0.771
Alcohol consumption	946 (36.1)	467 (40.5)	211 (35.6)	132 (31.6)	81 (30.5)	55 (28.6)	<0.001
Triage vitals, mean (SD)							
SBP, mmHg	191.4 (22.7)	189.1 (20.2)	190.9 (23.4)	191.9 (23.7)	197.4 (26.1)	198.8 (25.4)	<0.001
DBP, mmHg	108.1 (17.8)	107.4 (16.4)	108.6 (18.0)	108.7 (17.8)	109.5 (21.3)	107.0 (20.9)	0.163
Laboratory tests	3559 (98.9)	1611 (98.5)	820 (99.4)	555 (98.8)	335 (99.4)	238 (99.2)	0.181
Serum creatinine, mg/dL, mean (SD)	1.08 (1.18)	0.82 (0.48)	0.94 (0.61)	1.13 (0.92)	1.50 (1.56)	2.63 (3.10)	<0.001
eGFR, mL/min/1.73 m^2^, mean (SD)	80.1 (28.3)	89.6 (21.5)	82.8 (25.2)	74.1 (29.6)	63.2 (30.6)	47.4 (31.9)	<0.001
Troponin-I, ng/mL, mean (SD)	0.16 (1.99)	0.04 (0.25)	0.14 (1.61)	0.22 (1.20)	0.51 (5.38)	0.27 (1.59)	0.010
BNP, pg/mL, mean (SD)	354 (700)	149 (366)	279 (525)	421 (666)	569 (845)	945 (1260)	<0.001
Chest radiography done, *n* (%)	3393 (94.3)	1529 (93.5)	781 (94.7)	527 (93.8)	323 (95.8)	233 (97.1)	0.021
Cardiomegaly ^a^, *n* (%)	480 (14.1)	185 (12.1)	112 (14.3)	74 (14.0)	61 (19.0)	48 (20.6)	<0.001
ECG done, *n* (%)	3123 (86.8)	1417 (86.7)	696 (84.4)	469 (83.5)	313 (92.9)	228 (95.0)	0.001
LVH ^b^, *n* (%)	425 (13.6)	159 (11.2)	95 (13.7)	76 (16.3)	59 (19.0)	36 (15.8)	<0.001
Acute HMOD, *n* (%)	1427 (39.6)	566 (34.6)	326 (39.5)	222 (39.5)	176 (52.2)	137 (57.1)	<0.001

Data are presented as *n* (%) or mean (SD), as appropriate. SD, standard deviation; eGFR, estimated glomerular filtration rate; SBP, systolic blood pressure; DBP, diastolic blood pressure; BNP, B-type natriuretic peptide; ECG, electrocardiography; LVH, left ventricular hypertrophy; HMOD, hypertension-mediated organ damage; ^a^ Cardiomegaly on chest radiography was diagnosed when the ratio between the maximal horizontal cardiac diameter and the maximal horizontal inner thoracic cage diameter was > 0.5; ^b^ LVH on ECG was diagnosed when it satisfied either the Cornell voltage criterion (the amplitude of R in aVL plus the amplitude of S or QS complex in V3 with a cutoff of >2.8 mV in men and >2.0 mV in women) or the Sokolow–Lyon criterion (the amplitude of S in V1 plus the amplitude of R in V5 or V6 ≥ 3.5 mV).

**Table 2:** Outcomes of the index visits to the emergency department and during the follow-up period.

	All Patients (*n* = 3599)	Negative (*n* = 1635)	Dipstick Proteinuria				*p*-Value for Trend
			Trace (*n* = 825)	1+ (*n* = 562)	2+ (*n* = 337)	≥3+ *(n* = 240)	
Outcomes of the index ED visit, *n* (%)							
Admission	2199 (61.1)	869 (53.1)	515 (62.4)	367 (65.3)	259 (76.9)	189 (78.8)	<0.001
Discharge	1108 (30.8)	627 (38.3)	248 (30.1)	146 (26.0)	49 (14.5)	38 (15.8)	<0.001
Discharge against medical advice	288 (8.0)	139 (8.5)	62 (7.5)	48 (8.5)	28 (8.3)	11 (4.6)	0.176
Death in the ED	4 (0.1)	0 (0)	0 (0)	1 (0.2)	1 (0.3)	2 (0.8)	0.001
Revisit to ED, *n* (%)							
1-month revisits	267 (9.1)	109 (8.2)	60 (9.1)	50 (11.2)	33 (11.8)	15 (7.4)	0.193
3-month revisit	480 (16.4)	185 (13.9)	104 (15.7)	87 (19.5)	61 (21.8)	43 (21.1)	<0.001
1-year revisit	832 (28.4)	355 (26.7)	173 (26.1)	131 (29.3)	95 (33.9)	78 (38.2)	<0.001
Readmission, *n* (%)							
1-month readmission	150 (5.1)	62 (4.7)	33 (5.0)	27 (6.0)	20 (7.1)	8 (3.9)	0.354
3-month readmission	239 (8.2)	95 (7.1)	49 (7.4)	40 (8.9)	36 (12.8)	19 (9.3)	0.008
1-year readmission	364 (12.4)	146 (11.0)	72 (10.8)	54 (12.0)	55 (19.5)	37 (18.1)	<0.001
Mortality, *n* (%)							
1-month mortality	177 (4.9)	32 (2.0)	46 (5.6)	40 (7.1)	33 (9.8)	26 (10.8)	<0.001
3-month mortality	256 (7.1)	47 (2.9)	72 (8.7)	56 (10.0)	47 (13.9)	34 (14.2)	<0.001
1-year mortality	471 (13.1)	95 (5.8)	135 (16.4)	102 (18.1)	78 (23.1)	61 (25.4)	<0.001
3-year mortality	712 (19.8)	177 (10.8)	179 (21.7)	163 (29.0)	108 (32.0)	85 (35.4)	<0.001

Data are presented as *n* (%). ED, emergency department.

**Table 3:** Hazard ratios for mortality according to dipstick proteinuria among patients with hypertensive crisis.

Grade of Dipstick Proteinuria	Unadjusted	Model 1 *	Model 2 ^†^	Model 3 ^‡^
	HR (95% CI)	HR (95% CI)	HR (95% CI)	HR (95% CI)
Negative	REF	REF	REF	REF
Trace	2.19 (1.78–2.69)	1.91 (1.55–2.35)	1.86 (1.51–2.30)	1.91 (1.53–2.37)
(1+)	2.98 (2.41–3.69)	2.35 (1.90–2.91)	2.25 (1.81–2.80)	2.32 (1.85–2.91)
(2+)	3.45 (2.71–4.38)	2.49 (1.96–3.17)	2.38 (1.85–3.05)	2.40 (1.86–3.10)
(≥3+)	3.88 (3.00–5.03)	2.73 (2.10–3.54)	2.64 (2.01–3.46)	2.40 (1.78–3.24)

Data are presented as *n* (%). HR, hazard ratio; CI, confidence interval; REF, reference; * Model 1: Adjusted for age and sex; ^†^ Model 2: Adjusted for age, sex, systolic blood pressure, diastolic blood pressure, and comorbidities (hypertension, diabetes mellitus, ischemic stroke, hemorrhagic stroke, and coronary artery disease); ^‡^ Model 3: Adjusted for age, sex, systolic blood pressure, diastolic blood pressure, comorbidities (hypertension, diabetes mellitus, ischemic stroke, hemorrhagic stroke, and coronary artery disease), and components of hypertension-mediated organ damage (serum creatinine, cardiomegaly on chest radiography, and left ventricular hypertrophy on electrocardiography).

## Data Availability

The data that support the findings of this study are available from the corresponding author upon reasonable request.

## Supplementary Materials

The following supporting information can be downloaded at: https://www.mdpi.com/article/10.3390/jpm12060971/s1, Table S1: Principal diagnosis at the discharge of all patients.
