# Supplementary material for: Association of Dipstick Proteinuria with Long-Term Mortality among Patients with Hypertensive Crisis in the Emergency Department
Source: J Pers Med. 2022 Jun 14;12(6):971. doi: 10.3390/jpm12060971 (PMC9225554; doi:10.3390/jpm12060971)
Supplement: Supplementary file 1 [file jpm-12-00971-s001.zip › jpm-1763729-supplementary.pdf]

Supplementary Table S1. Principal diagnosis at discharge of all patients

	All patients (n=3,599)
Cardiovascular disorders	1,008 (28.0)
Neurologic disorders	990 (27.5)
Infectious diseases	464 (12.9)
Gastrointestinal disorders	389 (10.8)
Kidney disease	151 (4.2)
Respiratory disorders	132 (3.7)
Malignancies	87 (2.4)
Others	378 (10.5)

Data presented as n (%). Cardiovascular disorders included such as acute heart failure, angina pectoris, and acute myocardial infarction. Neurologic disorders included such as ischemic stroke, hemorrhagic stroke, and seizures. Infectious diseases included such as upper respiratory tract infection, lower respiratory tract infection, and urinary tract infection. Gastrointestinal disorders included such as gastroenteritis, gastrointestinal bleeding, and peptic ulcer. Respiratory disorders included such as asthma, chronic obstructive pulmonary disease, and pneumothorax. Kidney diseases included such as acute kidney injury, and progression of chronic kidney disease. Malignancies included all solid tumors and hematologic malignancies. Others included all diagnosis not classified above, included such as urinary tract stones, epistaxis, and benign paroxysmal positional vertigo.
